# Diagnostic Accuracy of Magnetic Resonance Enterography for the Evaluation of Active and Fibrotic Inflammation in Crohn’s Disease

**DOI:** 10.3389/fsurg.2022.872596

**Published:** 2022-05-13

**Authors:** Florian N. Loch, Carsten Kamphues, Katharina Beyer, Frederick Klauschen, Christian Schineis, Benjamin Weixler, Johannes C. Lauscher, Marc Dorenbeck, Christian Bayerl, Rolf Reiter

**Affiliations:** ^1^ Charité – Universitätsmedizin Berlin, corporate member of Freie Universität Berlin and Humboldt–Universität zu Berlin, Department of Surgery, Berlin, Germany; ^2^ Charité – Universitätsmedizin Berlin, corporate member of Freie Universität Berlin and Humboldt–Universität zu Berlin, Insitute for Pathology, Berlin, Germany; ^3^ Charité – Universitätsmedizin Berlin, corporate member of Freie Universität Berlin and Humboldt–Universität zu Berlin, Department of Radiology, Berlin, Germany; ^4^ Berlin Institute of Health at Charité – Universitätsmedizin Berlin, Berlin, Germany

**Keywords:** Crohn’s disease, magnetic resonance enterography, fibrosis, active inflammation, acute inflammation score

## Abstract

**Background:**

Despite the success of standard magnetic resonance enterography (MRE) in detecting Crohn’s disease (CD), characterization of strictures and, thus, therapy guidance is still limited. The aim of the study was to determine diagnostic accuracy of MRE in detecting or ruling out active inflammation and identifying fibrotic lesions in patients with terminal ileal CD with histopathology as reference.

**Methods:**

Sixty-seven consecutive patients (median age 32 years, range 19–79 years) with terminal ileal CD were retrospectively enrolled between January 2015 and October 2020. The median interval between MRE and surgery was 9 days (range 0–86 days). Sensitivity, specificity, positive and negative predictive value (PPV and NPV, respectively), and area under the curve (AUC) with 95% confidence intervals (CIs) were calculated for the MRE-based AIS (acute inflammation score) using the histopathology of surgical specimens as the reference standard.

**Results:**

Sensitivity, specificity, PPV, and NPV for detecting or ruling out active inflammation were 100% (CI, 0.94–1.00; 0.44–1.00; 0.93–1.00; 0.31–1.00) using an AIS cut-off of >4.1. AUC was 1.00 (CI, 1.00–1.00; *p* < 0.01). In all patients with fibrotic changes only and no active inflammation, AIS was <4.1. Interobserver agreement was substantial (*κ* = 0.65, *p* < 0.01).

**Conclusion:**

Our study has shown an excellent diagnostic performance of the MRE-based AIS for determining whether active inflammation is present or lesions are due to chronic changes in ileal CD using the histopathology of surgical specimens as reference. These findings indicate that the MRE-based AIS allows a better determination of the inflammatory stage of terminal ileal CD, which facilitates the decision to perform surgery.

## Introduction

Despite great advancements made in conservative anti-inflammatory therapy, 80% of patients with Crohn’s disease (CD) need at least one surgery during their lifetime (1, 2). Magnetic resonance enterography (MRE) is the most accurate imaging modality for detecting intestinal strictures in CD (3–5). While primarily fibrotic bowel strictures require endoscopy or surgery, primarily inflammatory strictures are treated by anti-inflammatory medication. Nevertheless, the diagnostic inability of standard MRE to accurately distinguish active inflammation from fibrosis facilitates the continuation of anti-inflammatory drug treatment instead of early surgery. Clinically, this can lead to persistent symptoms of intestinal obstruction with potential exacerbation and surgery shortly after high-dose immunosuppression. In these cases, the rate of early postoperative complications after eventual surgery, such as anastomotic leakage and the need for protective or even terminal stoma, is increased (6–10).

The acute inflammation score (AIS) has been suggested as a simple qualitative score to characterize inflammation in CD using ileal biopsy as reference (11). However, published data on the characterization of inflammation and fibrosis in CD lesions by MRE are limited because they were obtained in small study populations and used ileocolonoscopy or terminal ileal biopsy as the reference standard instead of a histopathological assessment of surgical specimens (11–17).

Therefore, the aim of our study was to investigate the diagnostic performance of MRE in detecting or ruling out active inflammation and identifying fibrotic lesions in ileal CD using the AIS and histopathology of surgical specimens as reference.

## Material and Methods

### Patients

**Figure 1:**
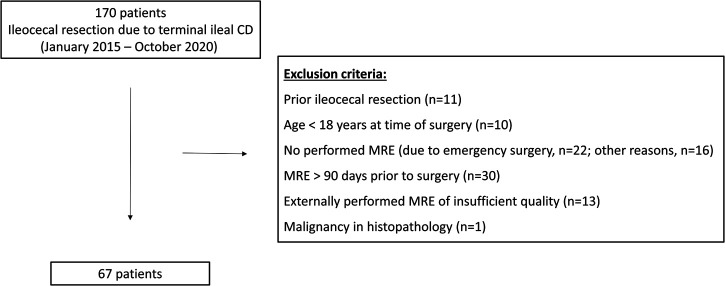
Flowchart of patient recruitment. The process of patient selection with the respective number of patients for each exclusion criterion is shown.

Our Institutional Review Board (#EA4/025/21) approved this retrospective, single-center study with written informed consent waived. Consecutive patients with proven terminal ileal CD who underwent ileocecal resection between January 2015 and October 2020 at the Department of Surgery, Campus Benjamin Franklin, Charité – Universitätsmedizin Berlin, Germany, were included. The inclusion criterion was preoperative MRE within 90 days prior to surgery. The exclusion criteria were resection of ileocolonic anastomosis after prior ileocecal resection, age <18 years at time of surgery, malignancy in histopathology, no preoperative MRE (e.g., in cases of emergency surgery or contraindication to MR imaging), MRE performed >90 days prior to surgery, and an externally performed MRE of insufficient quality for the purposes of the study. The process of patient selection with the respective number of patients excluded for each exclusion criterion is shown in Figure 1. Sixty-seven patients were included in this study, and their demographic and clinical characteristics are presented in Table 1.

**Table 1:** Demographic and clinical characteristics of the patient population investigated.

Patients	*n* = 67
Age	
Median age (years)	32
Age range (years)	19–79
Sex	
Female	41 (61.2%)
Male	26 (38.8%)
Time between initial diagnosis and surgery	
Median (years)	4
Range (years)	0–32
Medical therapy at time of surgery	
Corticosteroids	
Budesonide	6 (9.0%)
Budesonide and mesalazin	3 (4.5%)
Prednisone	8 (11.9%)
Prednisone and mesalazin	2 (3.0%)
Immunosuppressive agents (azathioprine, Mtx)	4 (6.0%)
Antibodies (adalimumab, infliximab, ustekinumab, vedolizumab)	10 (14.9%)
Corticosteroids and immunosuppressive agents	1 (1.49%)
Corticosteroids and antibodies	2 (3.0%)
Immunosuppressive agents and antibodies	7 (10.4%)
Corticosteroids and immunosuppressive agents and antibodies	5 (7.5%)
None	19 (28.4%)
Time between MRI and surgery	
Median (days)	9
Range (days)	0 (same day surgery)–86
Histopathological characteristics	
Active inflammatory terminal ileal CD	64 (95.5%)
Non-active inflammatory terminal ileal CD with chronic changes only	3 (4.5%)

### Magnetic Resonance Enterography and Assessment of Active Inflammation

MRE was performed at 1.5 Tesla (Magnetom Aera, Siemens Healthineers, Erlangen, Germany) or 3 Tesla (Magnetom Skyra, Siemens Healthineers, Erlangen, Germany) in accordance with ECCO guidelines (18). All images were retrospectively analyzed for the purposes of this study by two radiologists (RR, CB) with more than 5 years of experience in abdominal imaging, independently, and blinded to histopathology and patient history. The presence of active inflammation in terminal ileal CD was assessed using the AIS, which is based on the extent of mural thickness and mural edema (11). A qualitative score ranging from 0 to 3 was assigned as follows: mural thickness 0: 1–3 mm; mural thickness 1: >3–5 mm; mural thickness 2: >5–7 mm; mural thickness 3: >7 mm; and mural T2 signal 0: equivalent to a normal bowel wall; mural T2 signal 1: minor increase in the signal – the bowel wall appears dark gray on fat-saturated images; mural T2 signal 2: a moderate increase in the signal – the bowel wall appears light gray on fat-saturated images; mural T2 signal 3: a marked increase in the signal – the bowel wall contains areas of white, high signal approaching that of luminal content (11). It is calculated using the following formula: AIS = 1.79 + 1.34 mural thickness + 0.94 mural T2 score, and ranges from 1.79 to 8.63 (11). The established optimal AIS cut-off of >4.1 for the presence of active inflammation was used (11). Additionally, for the subgroup analysis of stricturing CD, all patients were assessed according to the CONSTRICT criteria (19) for imaging-based diagnosis of strictures as follows: (i) localized luminal narrowing with at least a 50% luminal diameter reduction, (ii) bowel wall thickening with a 25% increase in wall thickness relative to the adjacent nonaffected bowel, and (iii) prestricture dilatation greater than 3 cm.

### Surgery

All patients underwent laparoscopy-assisted or open ileocecal resection. Either primary ileocolonic anastomosis with or without diverting loop ileostomy or terminal ileostomy with closure of the colon was performed. As mentioned above, all patients with ileocolonic anastomosis after prior ileocecal resection were excluded from this study to focus on patients with terminal ileal CD with an intact ileocecal valve. The reasons for performing the surgery were symptomatic strictures of the terminal ileum in CD, limited, non-stricturing, ileocecal CD as an alternative to immunosuppressive therapy, and penetrating disease (abscess, fistula). In cases of stricturing disease due to active inflammation, surgery was predominantly performed in patients with persisting symptoms despite immunosuppressive therapy.

### Histopathological Assessment

For the purposes of this study, the original histopathological reports based on an analysis of formalin-embedded surgical specimens were reviewed. As the radiological AIS was assigned to the terminal ileum, a review of the histopathological reports focused on findings pertaining to the terminal ileal part of the specimens. Inflammation of the terminal ileum in each specimen was primarily characterized in terms of the presence or the absence of active inflammation. For this purpose, previously established histopathological criteria for the presence of active inflammation were used (11, 20). Based on these established criteria, the specimens of each patient were characterized as active inflammatory terminal ileal CD if erosion or ulceration, invasion of neutrophil granulocytes, cryptitis, and/or abscess formation were present. In the absence of any of these signs of histopathologically active inflammation, the specimens were classified as non-active inflammatory terminal ileal CD. Subsequently, in these cases of non-active inflammatory terminal ileal CD, histopathological reports were reviewed for confirmation of chronic inflammation and fibrosis. Therefore, as a reference standard for MRE assessment of active inflammation, the cases were subdivided into two histopathological groups: active inflammatory terminal ileal CD and non-active inflammatory terminal ileal CD with chronic changes only.

### Statistical Analysis

Sensitivity, specificity, positive and negative predictive value (PPV and NPV), and area under the curve (AUC) with a 95% confidence interval (CI) were calculated separately for each of the two readers. Interobserver agreement was calculated using Cohen’s kappa statistics. Associations between an AIS of > 4.1 and the presence of the histopathological signs of active inflammation were calculated using the *χ*^2^ test. The overall association of higher AIS values and the presence of the signs of histopathologically active inflammation was calculated using the Mann–Whitney *U*-test. The AUCs of the receiver operating characteristic curves of mural thickness and mural T2 signal were compared using the Delong method. The significance level was set to 5%. Data were analyzed using SPSS Statistics, Version 25.0 (IBM Corp. Released 2017. IBM SPSS Statistics for Windows, Version 25.0. Armonk, NY: IBM Corp.).

## Results

### Active Inflammation on Magnetic Resonance Enterography

**Figure 2:**
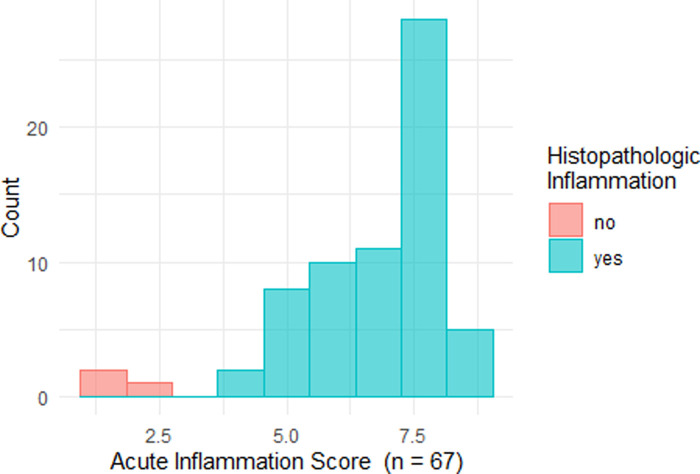
Histogram of acute inflammation scores. Histopathological findings are indicated by different colors: red – no signs of acute inflammation; turquoise: the presence of acute inflammation.

**Figure 3:**
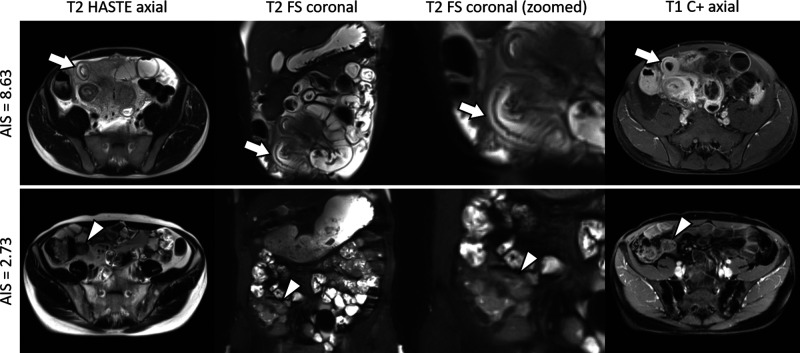
Representative cases. Upper row: A 37-year-old man with ileal Crohn’s disease and an increased acute inflammation score (AIS) of 8.63. The terminal ileum (arrows) shows the following imaging features of acute inflammation: (**A**) mural thickening of up to 9 mm; (**B**,**C**) a marked increase in the signal of the bowel wall consistent with edema; and (**D**) a marked contrast enhancement. Histopathology revealed severe ulcerative ileitis with active inflammation. Lower row: A 37-year-old man with ileal Crohn’s disease and a decreased AIS of 2.73. The terminal ileum (arrowheads) is characterized by the following findings: (**E**) no measurable mural thickening; (**F**,**G**) minor increase in the signal of the bowel wall indicating edema; and (**H**) minor contrast enhancement. Histopathology revealed focally dilated ileal wall with stromal fibrosis and no active inflammation. Note that supplementary contrast-enhanced images are shown to provide a comprehensive case overview, although they are not a part of the AIS assessment. HASTE, half-Fourier acquisition single-shot turbo-spin-echo sequence; FS, fat saturation; C+, contrast enhancement.

The distribution of AIS results is displayed in Figure 2. Figure 3 shows representative cases of increased (AIS > 4.1) and decreased (AIS < 4.1) inflammatory activity. Sixty-four patients showed signs of active inflammation on MRE (95.5%, AIS > 4.10), whereas three patients showed no signs of active inflammation (4.5%, AIS < 4.10). We found a median AIS of 6.75 with a range of 1.79–8.63.

### Comparison of Magnetic Resonance Enterography with Histopathology

For image analysis performed by the first radiologist, all 64 cases with active inflammatory terminal ileal CD had an AIS > 4.1 on MRE. The AIS was <4.1 in the three cases of non-active inflammatory terminal ileal CD with only chronic changes (two cases with fibrosis and one case with chronic granulomatous serositis). This corresponds to 100% sensitivity, specificity, PPV, and NPV (CI, 0.94–1.00; 0.44–1.00; 0.93–1.00; 0.31–1.00, respectively). The AUC was 1.00 (CI, 1.00–1.00; *p* < 0.01). The second radiologist achieved 95.3% sensitivity (CI, 0.87–0.98), 100% specificity (CI, 0.44–1.00), a PPV of 100% (CI, 0.93–1.00), and an NPV of 50% (CI, 0.14–0.86). AUC was 0.99 (CI, 0.97–1.00). Interobserver agreement was substantial (*κ* = 0.65, *p* < 0.01).

For the readings of both radiologists, there was a significant association between an AIS > 4.1 and the presence of histopathologically active inflammatory terminal ileal CD (*p* < 0.01) and an overall association of higher AIS values and the presence of histopathologically active inflammatory terminal ileal CD (*p* < 0.01).

Additionally, the diagnostic accuracy of mural thickness and the mural T2 signal were assessed separately. Compared with the AIS, accuracy was the same for mural thickness using an optimal cut-off of 3 mm (AUC of 1.00, CI, 1.00–1.00), whereas the mural T2 signal had a slightly lower accuracy, although the difference was not significant (*p* = 0.22; AUC of 0.93, 67% sensitivity, 100% specificity, a PPV of 100% and an NPV of 13%, CI, 0.82–0.92; 0.57–0.76; 1.00–1.00; 1.00–1.00; 0.03–0.25, respectively).

### Stricturing Crohn’s Disease

In our population of 67 patients, 32 cases (47.8%, mean AIS = 6.71 ± 1.77) met the imaging criteria for stricturing CD, whereas 35 cases (52.2%, mean AIS = 6.86 ± 1.09) did not. No significant difference was found for mean AIS with stricture vs. no stricture (*p* = 0.68). All of the cases with non-active inflammatory terminal ileal CD with chronic changes only in histopathology met the criteria for stricturing CD. A substratified analysis of the AIS in stricturing CD using histopathology as reference showed similar diagnostic accuracy for both readers: image analysis performed by the first radiologist resulted in 100% sensitivity, specificity, PPV, and NPV (CI, 0.88–1.00; CI, 0.29–1.00; CI,: 1.00–1.00; 1.00–1.00, respectively), and an AUC of 1.00 (CI, 1.00–1.00). Image analysis by the second radiologist resulted in 96.6% sensitivity (CI, 0.82.2–0.99), 100% specificity (CI, 1.00–1.00), a PPV of 100% (CI, 1.00–1.00), NPV of 75% (CI, 0.30–0.95), and an AUC of 0.98 (CI, 0.94–1.00). Interobserver agreement was almost perfect (*κ* = 0.84, *p* < 0.01).

Moreover, in the subgroup of stricturing CD (*n* = 32), there was a significant difference for patients with histopathologically active inflammatory terminal ileal CD (*n* = 29, a mean AIS of 7.2 ± 0.96) vs. patients with non-active inflammatory terminal ileal CD with chronic changes only (*n* = 3, a mean AIS of 2.1 ± 0.44, *p* < 0.001).

## Discussion

The results of our retrospective single-center study show that the MRE-based AIS can confirm or rule out active inflammation in terminal ileal CD and identify non-active inflammatory terminal ileal CD with only chronic changes with an excellent diagnostic performance using the histopathology of surgical specimens as reference.

The AIS was introduced by Stewart et al. in a study of 16 CD patients as derivation cohort and terminal ileal biopsy as reference. They achieved an AUC of 0.77 for predicting active inflammation in a validation cohort of 26 patients (11). In our study, we found a higher accuracy of the AIS (an AUC of 1.00), which might be attributable to the fact that we investigated a surgical population of patients who tend to have advanced disease. Our assessment of mural thickness and T2 signal intensity as separate test parameters revealed the same accuracy for mural thickness (an AUC of 1.00) and a slightly lower accuracy for the mural T2 signal (an AUC of 0.93). These findings suggest that the assessment of wall thickness *or* edema would suffice to identify active inflammation, rendering the calculation of the AIS redundant. However, this is a preliminary result that needs to be carefully validated.

Another result of our study is that there was no significant difference in mean AIS between patients with and without stricturing CD, while all of the histopathologically exclusive fibrotic lesions were associated with strictures. Moreover, in the subgroup of stricturing CD, there was a significant decrease in mean AIS for patients with exclusively fibrotic lesions vs. patients with active inflammation. This facilitates and objectifies the decision to consider surgery in patients with imaging criteria for strictures and a lower AIS. Sinha et al. investigated the detection of abnormal bowel segments in 49 CD patients using MRE and the histopathology of surgical specimens (21). Similar to our results, the authors found a high AUC value for mural thickness (an AUC of 0.96), whereas the diagnostic performance of the enhancement ratio of the abnormal bowel (an AUC of 0.81) and of the mesentery enhancement ratio (an AUC of 0.68) was moderate. Rimola et al. investigated 41 CD patients, also using MRE and the histopathology of surgical specimens. They found fibrosis to be correlated with the relative increase in enhancement, which discriminated mild and moderate from severe fibrosis with a high AUC of 0.93 (13). Rimola et al. also introduced the Magnetic Resonance Index of Activity score, which combines contrast enhancement, wall thickness, and the presence of edema and ulcers. The authors investigated 50 CD patients using ileocolonoscopy as reference and found a high AUC of 0.89 (16, 22). In the future, MRI techniques such as magnetization transfer MRI and MR elastography might potentially be used for a quantitative assessment of CD (23, 24).

This study has limitations. First, the retrospective study design did not allow a precise matching of the histopathology and MRE findings. However, we were able to minimize this limitation as we could derive the histopathological information regarding inflammation of the terminal ileal part of the resected specimen from the original histopathological reports in all cases included in this study. Second, our study population included only three cases showing a complete absence of the histopathological signs of active inflammation with chronic lesions only. This is due to the intention to have a clear separation of both groups and prevent an assessment of subgroups with mixed inflammation and fibrosis in histopathology without the possibility of exact quantification due to retrospective study design. Therefore, future studies should investigate the diagnostic performance of MRE with a histopathological quantification of inflammation and fibrosis as reference. For this purpose, histopathological parameters and scoring systems to distinguish the level of active inflammation and fibrosis for an exact quantification of the respective type of inflammation need to be further investigated (25). Thus, in addition to patients with exclusively fibrotic strictures, also those with predominantly fibrotic strictures could be identified, a subgroup of patients that would also benefit from quicker referral to surgery. Third, all patients in our study population underwent surgery, which preselects patients with advanced disease. More precisely, patients referred to surgery often have pronounced fibrotic strictures or an advanced degree of active inflammation that is therapy-refractory and regularly accompanied by penetrating disease. This has probably led to an overestimation of the diagnostic performance of identifying the respective characteristic of a lesion using a surgical cohort. Nevertheless, endoscopic assessment and biopsy are only superficial and cannot detect transmural disease, whereas a histopathological assessment of surgical specimens is the best reference standard currently available (19). The strengths of our study are the comparably large study population, the histopathology of surgical specimens as reference, and a short interval between MRE and surgery.

Acknowledging these limitations, we showed an excellent diagnostic accuracy for AIS of the terminal ileum in identifying patients with non-active inflammatory terminal ileal CD who only have chronic lesions. This group of patients would benefit from upfront surgery instead of continuing or even intensifying anti-inflammatory treatment. Such a strategy would lead to early relief of symptoms and avoid a higher rate of early postoperative complications after eventual surgery shortly after intensified immunosuppression.

In conclusion, our results show that the MRE-based AIS can demonstrate or rule out active inflammation in terminal ileal CD and identify cases with non-active inflammatory terminal ileal CD with only chronic lesions with an excellent diagnostic performance in a surgical cohort using the histopathology of surgical specimens as reference. This provides a more detailed insight into the nature of the inflammatory stage of intestinal strictures, with the potential to facilitate the decision to initiate surgical treatment. Prospective studies with comprehensive MRE mapping and exact histopathological cross-reference using surgical specimens are warranted.

## Data Availability

The datasets used and/or analyzed during the current study are available upon reasonable request pending approval by the local data security authorities.
